# The most appropriate titanium mesh cage size for anterior spinal reconstruction after single-level lumbar total en bloc spondylectomy: a finite element analysis and cadaveric validation study

**DOI:** 10.1186/s13018-021-02326-4

**Published:** 2021-03-09

**Authors:** Permsak Paholpak, Winai Sirichativapee, Taweechok Wisanuyotin, Weerachai Kosuwon, Yuichi Kasai, Hideki Murakami

**Affiliations:** 1 Department of Orthopedics, Faculty of Medicine, Khon Kaen University, Khon Kaen, 40002 Thailand; 2 Musculoskeletal Oncology Research Group, Khon Kaen University, Khon Kaen, Thailand; 3 Department of Orthopedic Surgery, Graduate School of Medical Sciences, Nagoya City University, Nagoya, Japan

**Keywords:** Total en bloc spondylectomy; TES, Finite element model, Construct rigidity, Anterior Reconstruction, Titanium mesh cage diameter

## Abstract

**Purpose:**

There is little information available regarding the cage diameter that can provide the most rigid construct reconstruction after total en bloc spondylectomy (TES). The aim of this study was thus to determine the most appropriate titanium mesh cage diameter for reconstruction after spondylectomy.

**Methods:**

A finite element model of the single level lumbar TES was created. Six models of titanium mesh cage with diameters of 1/3, 1/2, 2/3, 3/4, 4/5 of the caudad adjacent vertebra, and 1/1 of the cephalad vertebra were tested for construct stiffness. The peak von Mises stress (MPa) at the failure point and the site of failure were measured as outcomes. A cadaveric validation study also conducted to validate the finite element model.

**Results:**

For axial loading, the maximum stress points were at the titanium mesh cage, with maximum stress of 44,598 MPa, 23,505 MPa, 23,778 MPa, and 16,598 MPa, 10,172 MPa, 10,805 MPa in the 1/3, 1/2, 2/3, 3/4, 4/5, and 1/1 diameter model, respectively. For torsional load, the maximum stress point in each of the cages was identified at the rod area of the spondylectomy site, with maximum stress of 390.9 MPa (failed at 4459 cycles), 141.35 MPa, 70.098 MPa, and 88.972 MPa, 42.249 MPa, 15.827 MPa, respectively. A cadaveric validation study results were coincided with the finite element model results.

**Conclusion:**

The most appropriate mesh cage diameter for reconstruction is 1/1 the diameter of the lower endplate of the adjacent cephalad vertebra, due to its ability to withstand both axial and torsional stress. According to the difficulty of large size cage insertion, a cage diameter of more than half of the upper endplate of the caudad vertebrae is acceptable in term of withstand stress. A cage diameter of 1/3 is unacceptable for reconstruction after total en bloc spondylectomy.

## Introduction

Total en bloc spondylectomy (TES) is a procedure aimed at total removal of spinal tumors [1, 2]. Successful TES results in a lower local recurrence rate and better prognosis in primary spinal tumor and secondary spinal metastasis patients [3–7]. However, achieving total resection with TES also results in structural spinal nstability, which requires circumferential reconstruction using pedicular screws and rods and anterior column reconstruction using an autologous bone graft [2, 8–10]. Even with rigid stabilization and fixation, hardware failure is a late post-operative complication in TES. Bandiera et al. reported a 7% hardware failure in TES patients and proposed that it might be due to short segment fixation and late imbalance of the spinal column. Park et al. found that 37.5% of TES patients experienced titanium rod fracture, which occurred at an average of 29.2 months and that lumbar location and history of radiation therapy were risk factors for implant failure [11].

An expandable titanium cage, titanium mesh cage, and allogenous strut bone graft are options for anterior spinal column support in reconstruction after TES [3, 7, 9, 11–13]. The study by Park et al. mentioned above also found that the mode of anterior column reconstruction was not significantly related to rod fracture [11]. Even less common, the anterior cage breakage or cage subsidence can be occurred and usually required revision surgery [14–16]. To the best of our knowledge, there is limited information available regarding the appropriate implant diameter to be used in anterior spinal column reconstruction after TES.

The objective of this study was to determine the most appropriate diameter of titanium mesh cage to be used in anterior spinal column reconstruction after single-level lumbar TES surgery.

## Methods

A finite element model (FEM) of the lumbar spine was created using a normal CT of the lumbar spine. A Young’s modulus of 17,000 MPa and Poisson’s ratio of 0.3 was applied to all vertebrae cortical bone. For cancellous bone and intervertebral disk, a Young’s modulus of 100 MPa and 7.5 MPa and Poisson’s ratio of 0.2 and 0.4 were applied in order.

**Fig. 1:**
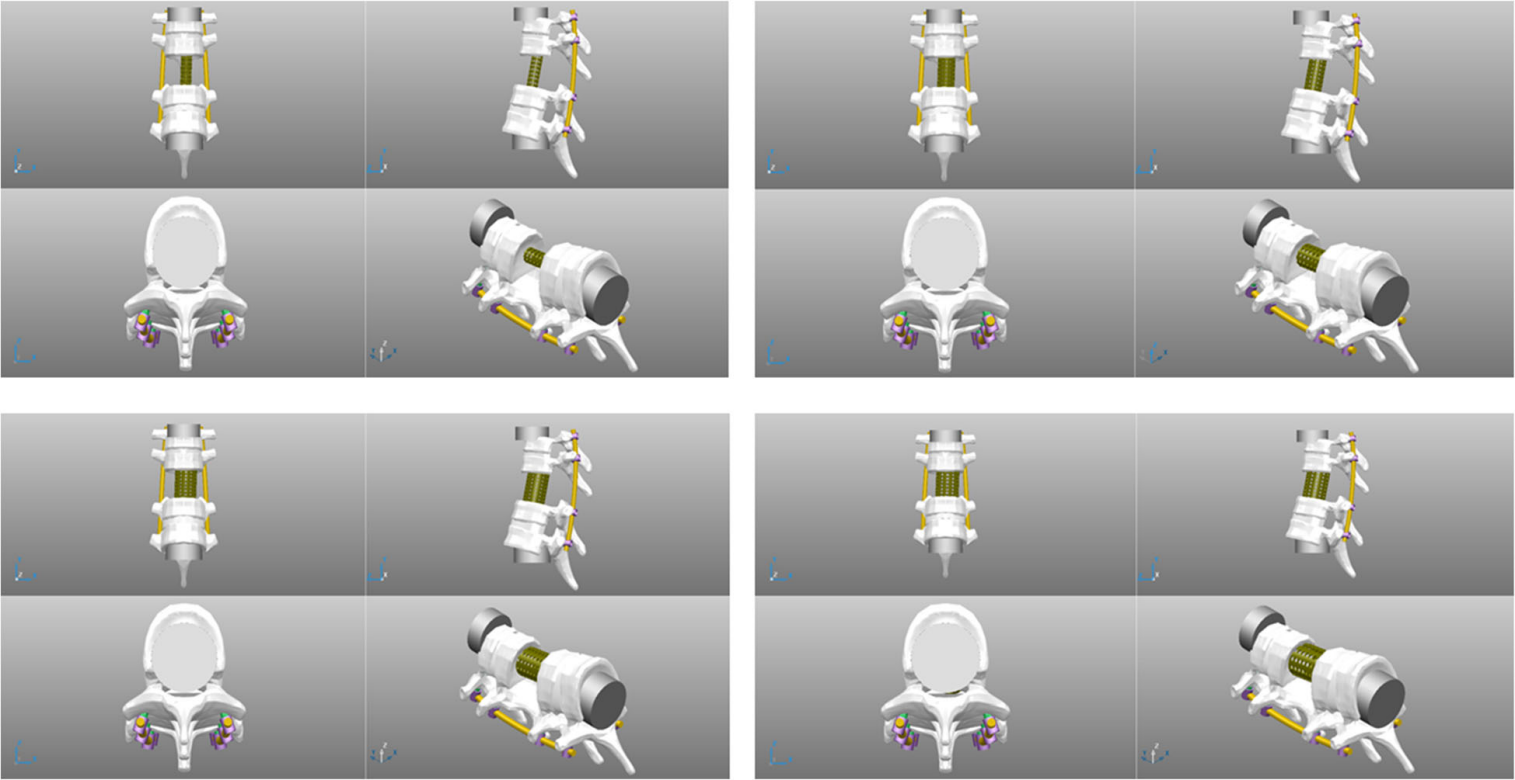
The finite element of four titanium mesh cage diameter models in single third lumbar spine total en bloc spondylectomy (TES)

The third lumbar vertebra and the adjacent intervertebral disks were removed to imitate a single level TES model. Models of six diameters of titanium mesh cage (1/3, 1/2, 2/3, 3/4, 4/5 of the diameter of the caudad vertebra), and 1/1 of the diameter of the cephalad vertebra were created using a Young’s modulus of 35,000 MPa and Poisson’s ratio of 0.36 as Akamaru et al. [17]. Pedicle screws of 6 mm in diameter and 45 mm in length and a rod of 5.5 mm in diameter were created using the Young’s modulus of 110,000 MPa and suing the same Poisson’s ratio as the same as the titanium mesh cage (Fig. 1). The screws were securely inserted into the pedicle and vertebral bodies of two spinal levels above and below on both sides and the rods were created to securely attach to all pedicle screws on both sides as a single unit. All material of each was set to be isotropic and homogenous. We decided to use the reconstruction construct of 2 levels above and below spondylectomy according to the previous literatures [8, 9, 17].

The finite element models were created using ANSYS 14.5 (Ansys Inc., Canonsburg, Pennsylvania, USA).

We tested each model for force in both the axial and torsional load directions. The axial load was applied to each model until it reached the failure point, which was defined as the point at which the instrumentation system or spinal unit moved more than 3 mm in any direction. The maximum stress (MPa) and maximum stress point were recorded. The axial load force was set at 1000 N. Torsional stress of 5 N-m was used to test at each model and applied until the failure point was reached. The peak von Mises stress value (MPa) of each model was recorded.

## Results

The results of the compression and rotation loads in all four models are shown in Table 1.

**Table 1:** The peak von miles stress under the compression and torsional load to system failure

Model of titanium mesh cage	Peak von Mises stress under compression load to failure (MPa)	Peak von Mises stress under torsional load to failure (MPa)
Diameter 1/3	44,598	390.9 (break at 4459 cycles)
Diameter 1/2	23,505	141.35 (>1 million cycle)
Diameter 2/3	23,778	70.098 (>1 million cycle)
Diameter 3/4	16,598	88.972 (>1 million cycle)
Diameter 4/5	10,172	42.249 (>1 million cycle)
Diameter 1 (same diameter as cephalad vertebra)	10,805	15.827 (>1 million cycle)

*MPa* megapascal

The 1/1 diameter cage exhibited the greatest stiffness under an axial compression load of 1000 N until failure. The 4/5 cage diameter also performed well under axial compression load. The 1/3 diameter cage was the weakest model, and the 1/2 and 2/3 diameter models performed similarly.

The 1/3 diameter model failed at a von Mises stress value of 390.9 with only 4459 cycles of 5 N-M torsional load. The 1/1 diameter model exhibited the greatest stiffness under torsional load. The 2/3 diameter cage exhibited greater stiffness when compare to 3/4 diameter cage and the Von Mises stress value was similar to that of the 3/4 diameter model.

**Fig. 2:**
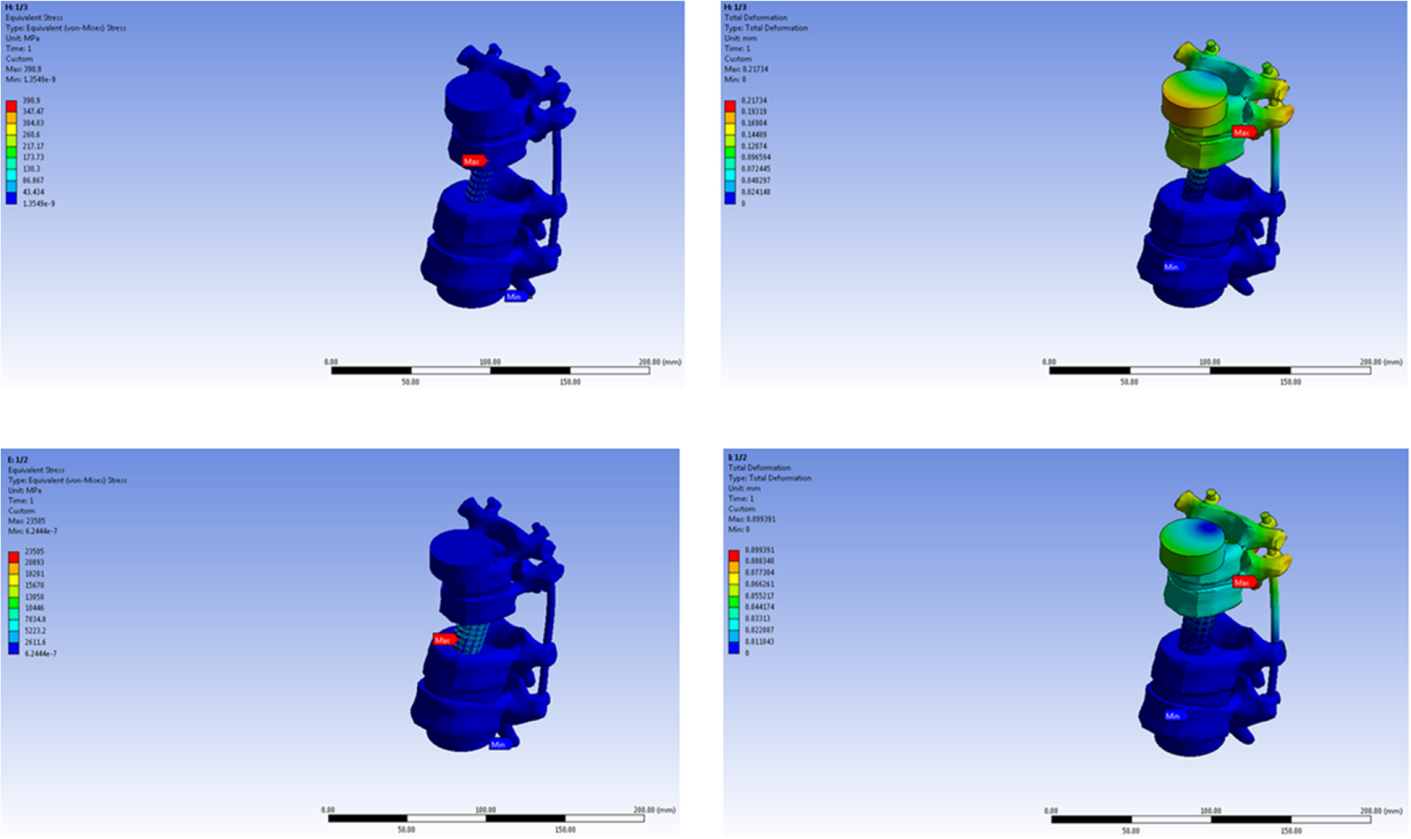
The maximum stress point on finite element model of 1/3 and 1/2 titanium mesh cage diameter model under axial and torsional load

**Fig. 3:**
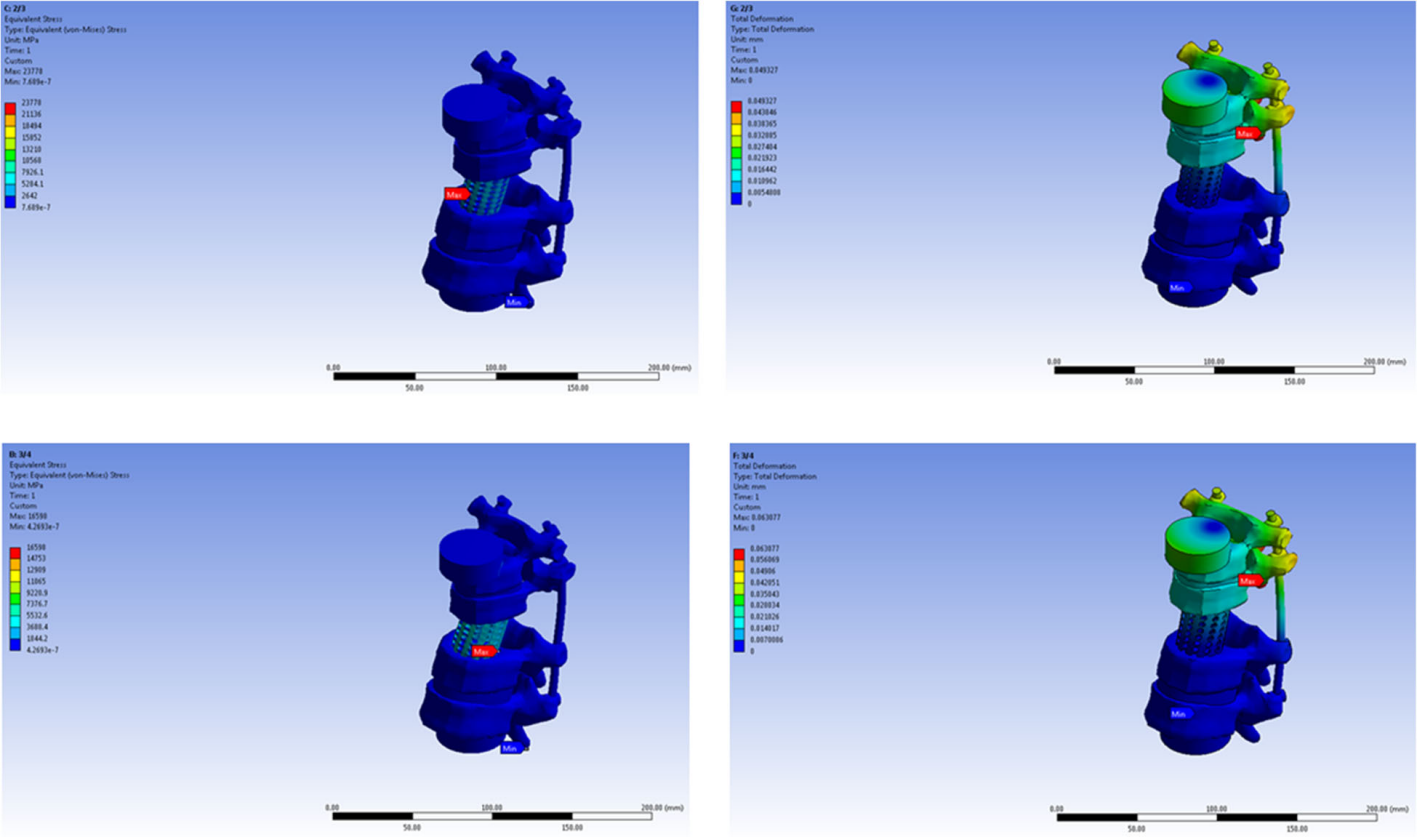
The maximum stress point on finite element model of 2/3 and 3/4 titanium mesh cage diameter model under axial and torsional load

**Fig. 4:**
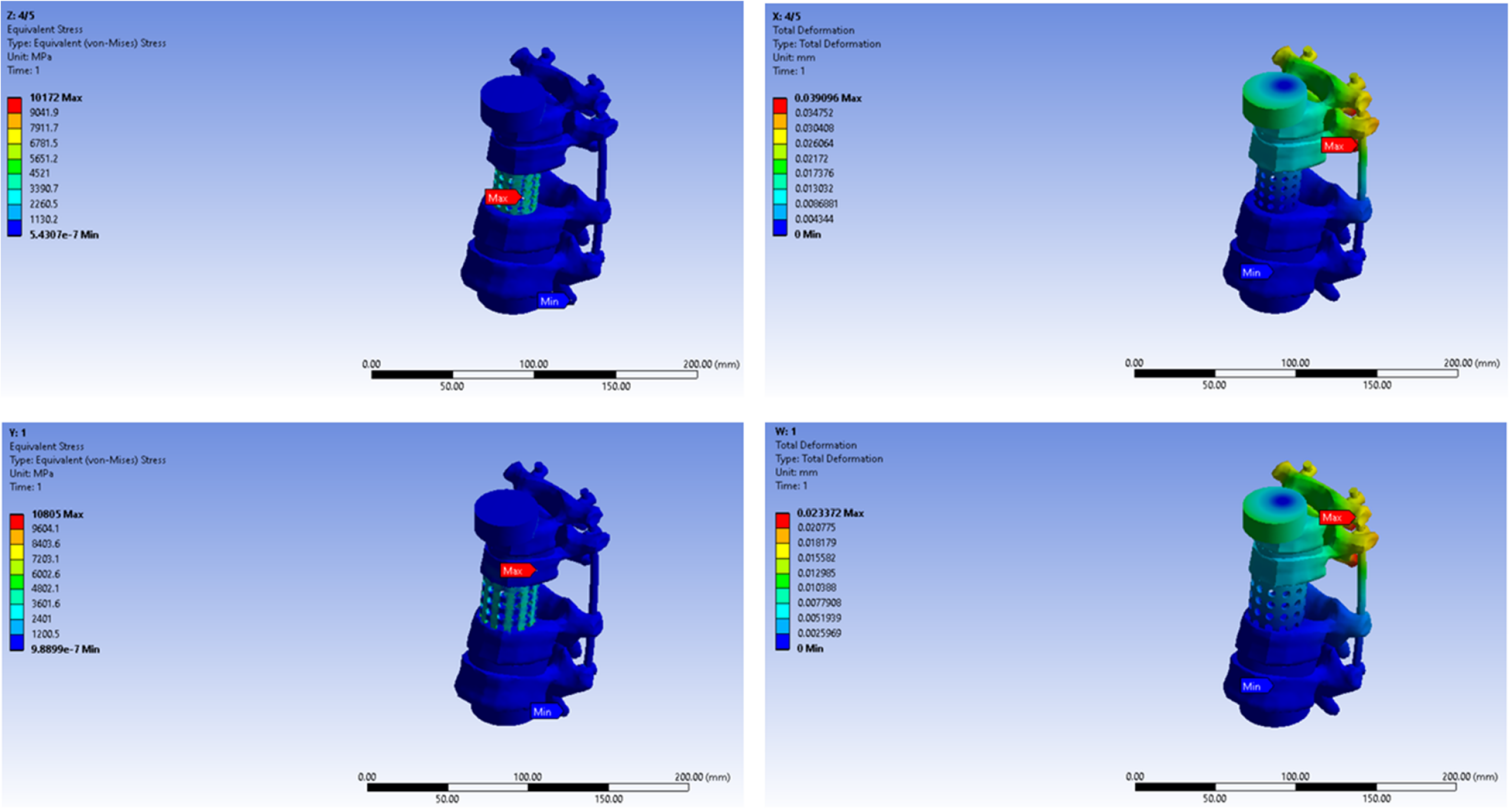
The maximum stress point on finite element model of 4/5 and 1/1 titanium mesh cage diameter model under axial and torsional load

The maximum stress load point, under axial compression load was at the upper-anterior part of the titanium mesh cage in the 1/3 diameter model, lower-anterior part of the titanium mesh cage in the 1/2 diameter model and 2/3 diameter models, at the lower-posterior part of the titanium mesh cage in the 3/4 and 4/5 diameter model, and at the cephalad vertebra in 1/1 model. The maximum stress point under torsional load in all models was at the rod just below the cephalad adjacent vertebra pedicle screw in all models except the 1/1 model (Figs. 2, 3, and 4).

The additional cadaveric experiments using Lumbar spines (L-spine) of a human cadaver were conducted to evaluate the validity of the FEM model as well as our research results’ credibility.

**Fig. 5:**
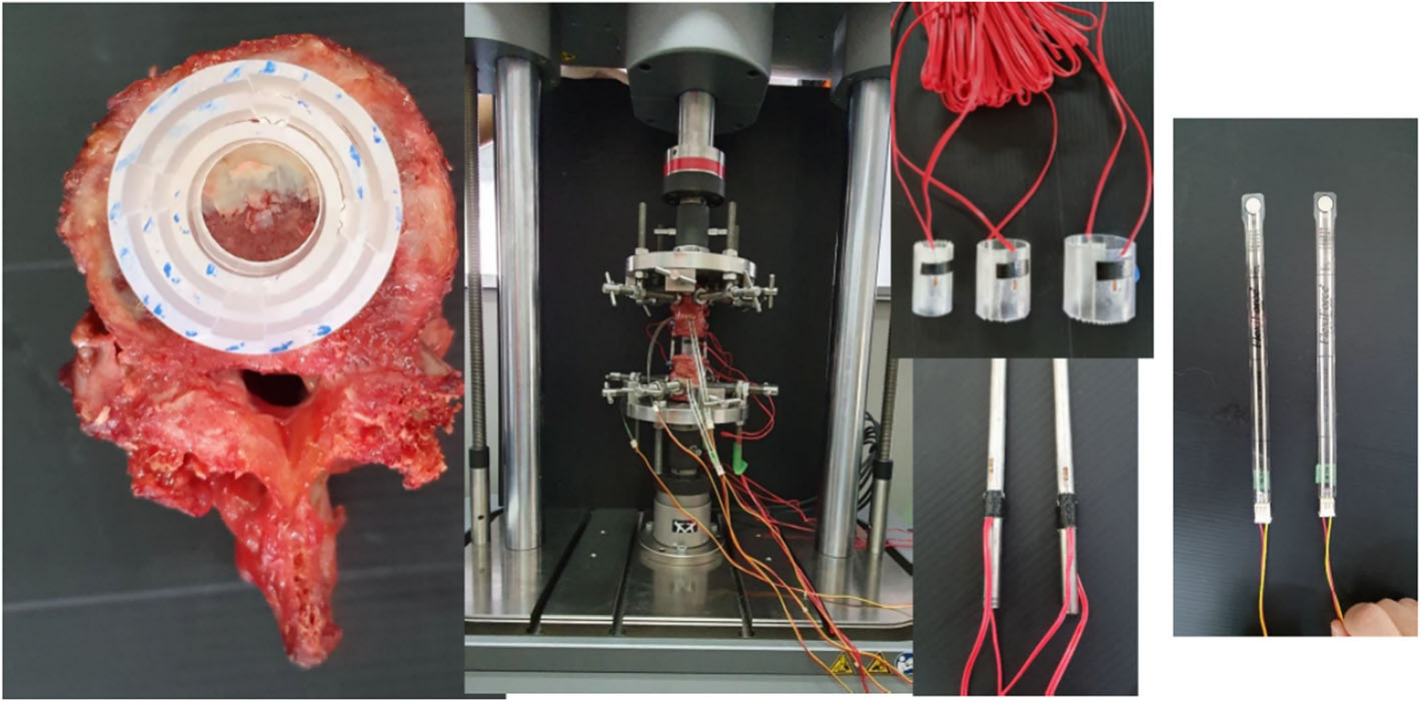
Cadaveric validation test of various diameter cage diameter models using Instron’s Electropuls E10000TM (Grove City, Pennsylvania, USA) machine. The stain gages were attached to the rods

After removed, the first to the fifth L-spines from a fresh cadaver (a 51-year-old female, cause of death: cardiac arrest) stored at Khon Kaen University, and constructed a model simulating a case of total spondylectomy of the third L-spine. We fixed L-spines with a pedicle screw system (Suiren® system by KiSCO CO., LTD., Kobe, Japan), then constructed three cylindrical spinal cages (3 cm in height with three different diameters; one-third, half, and two-third of the fourth L-spine width) with photo-curing resins (SCR735), and attached strain gages in front and back at the center of right and left rods as well as in front and back at the center of each cage (Fig. 5).

As the test device, Instron’s Electropuls E10000TM (Grove City, Pennsylvania, USA) was used. We then conducted compression tests under the initial compression load of 100 N up to 500 N (with the compression speed of 0.1mm/s) as well as rotation tests under 5 Nm rotation force (with the rotation speed of 0.1 deg/s) three times to obtain the amount of change in strain on the rods of both sides and on each cage in the third tests, and compared the results with the FEM data.

As for the compression test results, the amount of change in strain on spinal cages showed −2578 με at front/−1823 με at the back in the test using one-third width cage, −1441 με at front/−424με at the back with the half-width cage, and −851με at front/−141με at the back with using the two-third width cage, respectively, indicating a decreasing tendency in strain amount according to the increase of the cage diameter.

**Fig. 6:**
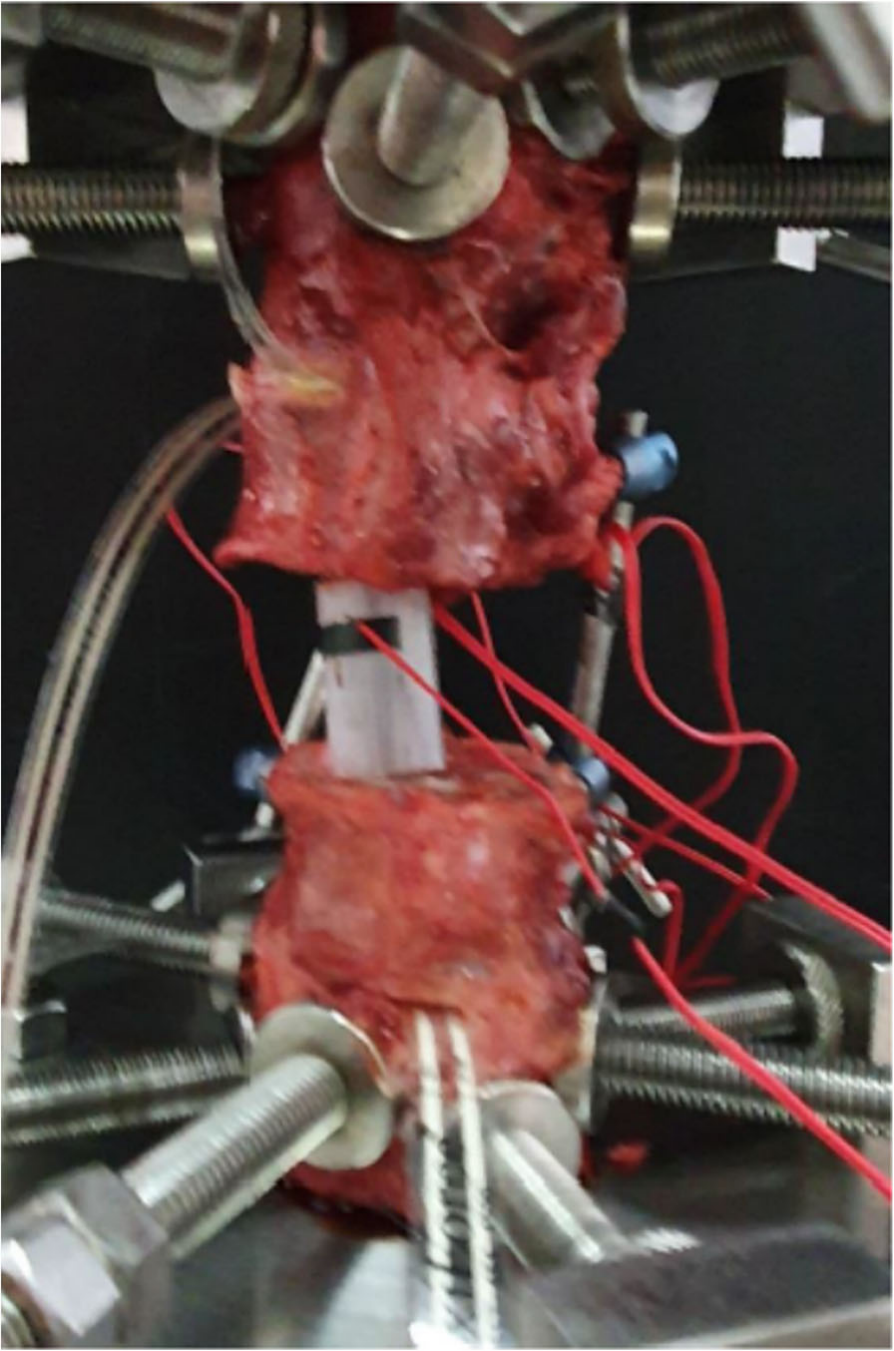
The 1/3 diameter cage dislodged after sustained rotation loaded test

As for the rotation test results of the amount of change in strain on rods showed 1356 με at front/−1250 με at the back in the right side rod and 913με at front/−875 με at the back in the left side rod in the test using the one-third width cage, showing cage dislodgement during the loading test (Fig.6). Regarding the results of the other tests, the change amount in strain showed 373 ε at front/−472 με at the back in the right side rod and 187 με at front/−102 με at the back in the left side rod in the test using the half-width cage, 28 μμε at front/−80με at the back in the right side rod, and 160 με at front/−135 με at the back in the left side rod with the two-third width cage, respectively. We did not confirm cage dislodgement in these tests, which also demonstrated the decreasing tendency in strain amount according to the increase of the cage diameter .

These validation tests showed a tendency coinciding with the FEM results, demonstrating the validity of the FEM model as well as the credibility of the FEM analysis in our present study.

## Discussion

In order to achieve stable fixation and bone fusion, the reconstruction method of choice is two cephalad and caudad spinal levels adjacent to TES level pedicle screws and rod fixation adjunct with anterior spinal reconstruction with cage [17].

Anterior spinal reconstruction is one of the most important steps to achieve stable fixation after TES [9, 14], and titanium mesh and expandable cages are commonly used implants for anterior spinal support [2, 3, 7–9, 12, 18, 19]. The advantages of the titanium mesh cage are that it varies in diameter and height, allows for more space for mercerized autogenous bone graft, is resistant to subsidence, and enables stable spinal reconstruction.

The most common implant-related failure after TES is breakage of the pedicular screws and rods system [3, 10, 11, 14], which can come under greater stress in cases of titanium mesh cage failure. The mechanical strength of the titanium mesh cage depends on its diameter and the biomaterial. Our finite element study found that the larger diameter the stiffer resistance to load. The 1/1 and 4/5 models showed the stiffest resistance to both axial compression and torsional load. The 2/3 diameter cage exhibited the stiffer resistance to compression load than the 3/4 and 1/2 models. Against torsional load, beside 4/5 and 1/1 model, the 2/3 diameter cage was the stiffest. Under torsional load, failure of the construct only occurred in the 1/3 diameter cage. In addition, we found that the maximum stress point under compression load was the titanium mesh, suggesting that it is susceptible to breakage after axial compression stress. However, under torsional load, stress was greatest at the rod, making rod breakage the greatest concern in these cases.

Even the 1/1 and 4/5 models showed the best results in term of stiffness under axial compression and torsional load. The insertion of the large titanium mesh cage in between the vertebrae at the spondylectomy during operation was difficult and had the risk of impingement to the thecal sac. The smaller cage, easier for insertion, the 2/3 and 3/4 diameter cages exhibited the appropriate stiffness under axial and torsional load. The 1/2 diameter cage was able to bear nearly the same compression load as the 2/3 diameter cage but was weaker against torsional load.

Regarding cage dislodgement under torsional load, the instantaneous axis of rotation (IAR) in the spine of healthy subjects in normal conditions is usually posterior to the anulus or near the center of the anterior spinal canal [20, 21]. When the pedicle screw system is inserted, however, the IAR would move backward, close to the middle point of the spinal rods on the left and right sides [22]. Therefore, when the distance between IAR and the spinal cage, or so-called lever arm length should be extended, relatively stronger rotational torque would be applied to cages, which should possibly lead to cage dislodgement if the cage itself is not stable enough. Thus, the cage diameter should be a vital factor to consider in the sense of cage stability.

Based on the above results, the 4/5 and 1/1 diameter cages are recommended to be used as the implant of choice to achieve rigid spinal reconstruction for both anterior and posterior approaches of TES. But very big cages cannot be used for only posterior approach of TES due to some difficulties of surgical procedures. Thus, if the larger-diameter cages are not available (which is often the case in developing countries), a 2/3 and 3/4 diameter titanium mesh cages are also acceptable for use as an anterior spinal column reconstruction device after TES. The 1/2 diameter cage is least diameter which acceptable to use after spondylectomy.

This study was limited in that (a) it was based on a single level lumbar spine finite element model which might not account for thoracic spine and (b) that further cadaveric biomechanical study is needed.

In conclusion, a titanium mesh cage with a diameter of more than half vertebral body diameter can withstand compression-load and torsional-load stress without construction failure. Smaller cages should not be used in anterior column reconstruction after single-level TES in the lumbar spine.

## Data Availability

Not applicable.

## Abbreviations

TES: Total en bloc spondylectomy
FEM: Finite element model
